# Analysis of D-dimer levels for the detection of deep venous thrombosis for patients with spinal metastasis undergoing decompression with fixation

**DOI:** 10.1186/s12891-024-07792-5

**Published:** 2024-08-27

**Authors:** Yun-qi Jiang, Yu-zhu Wang, An-nan Hu, Jian Zhou, Xi-lei Li, Qing Qi, Jian Dong

**Affiliations:** 1 Department of Orthopaedic Surgery, Zhongshan Hospital, Fudan University, 180 Fenglin Road, Shanghai, 200032 PR China; 2 Department of Pharmacy, Zhongshan Hospital, Fudan University, Shanghai, PR China; 3 Department of Ultrasonography, Zhongshan Hospital, Fudan University, 180 Fenglin Road, Shanghai, 200032 PR China

**Keywords:** Spinal metastasis, D-dimer, Deep venous thrombosis, Venous thromboembolism, Receiver operating characteristic

## Abstract

**Background:**

Deep venous thrombosis (DVT) after spinal surgery has recently attracted increasing attention. Patients with spinal metastases who undergo decompression with fixation are at a high risk of developing DVT. D-dimer levels indicate the risk of DVT, and the purpose of our study was to investigate D-dimer levels as a predictor of DVT perioperatively.

**Methods:**

We prospectively evaluated 100 patients with spinal metastases. D-dimer tests were performed twice: once before surgery and one day postoperatively. DVT was diagnosed by duplex ultrasonographic assessment of both lower extremities. Pulmonary embolisms (PEs) were diagnosed using multidetector computed tomography and pulmonary angiography. Perioperative serum D-dimer levels were compared between the DVT (+) and DVT (-) groups. The cutoff value of the D-dimer level was calculated using receiver operating characteristic analysis.

**Results:**

Preoperative and postoperative DVT prevalences were 8.0% (8/100) and 6.6% (6/91), respectively, and none of the patients developed PE. Before surgery, there was no significant differences in D-dimer levels between the pre-DVT (+) and pre-DVT (-) groups. After surgery, the D-dimer level one-day postoperatively for the post-DVT (+) group (17.6 ± 11.8 mg/L) was significantly higher than that of the post-DVT (-) group (5.0 ± 4.7 mg/L). The cutoff value of the postoperative D-dimer level was 9.51(mg/L), and the sensitivity and specificity for the optimum threshold were 83.3% and 89.4%, respectively.

**Conclusions:**

Our findings suggest that preoperative D-dimer level may not be a predictor of DVT. Preoperative ultrasound examinations should be routinely performed in patients with spinal metastases. Postoperative D-dimer levels greater than 9.51(mg/L) are a predictive factor for the early diagnosis of DVT after spine surgery.

**Trial registration:**

Our study was registered on Chinese Clinical Trial Registry (No.ChiCTR2000029737). Registered 11 February 2020 - Retrospectively registered, https://www.chictr.org.cn/index.aspx.

**Supplementary Information:**

The online version contains supplementary material available at 10.1186/s12891-024-07792-5.

## Background

Venous thromboembolism (VTE), including deep venous thrombosis (DVT) and pulmonary embolism (PE), is a fatal complication of spinal surgery [1]. DVT can progress to PE, and the reported rates of PE after spinal surgery range from 0 to 7.6%, while those of DVT range from 0.27–31% [2–4]. The variation in the data indicates that some patients may have characteristics that predispose them to VTE [5]. Moreover, malignant tumors are considered high-risk factors for DVT [6], and patients with spinal metastases undergoing nerve decompression with internal fixation are at a high risk for DVT. Clinically, DVT may occur before surgery [7]; therefore, the predictive diagnosis of DVT is crucial.

Many researchers have reported that D-dimer levels are indicative of a high risk of DVT or PE [8–12]. However, few studies have assessed the accuracy of DVT detection in patients with spinal metastases undergoing decompression with fixation. Therefore, the purpose of our study was to investigate the prevalence of DVT and PE and the usefulness of D-dimer levels as a perioperative predictor of DVT.

## Methods

### Study design

**Fig. 1:**
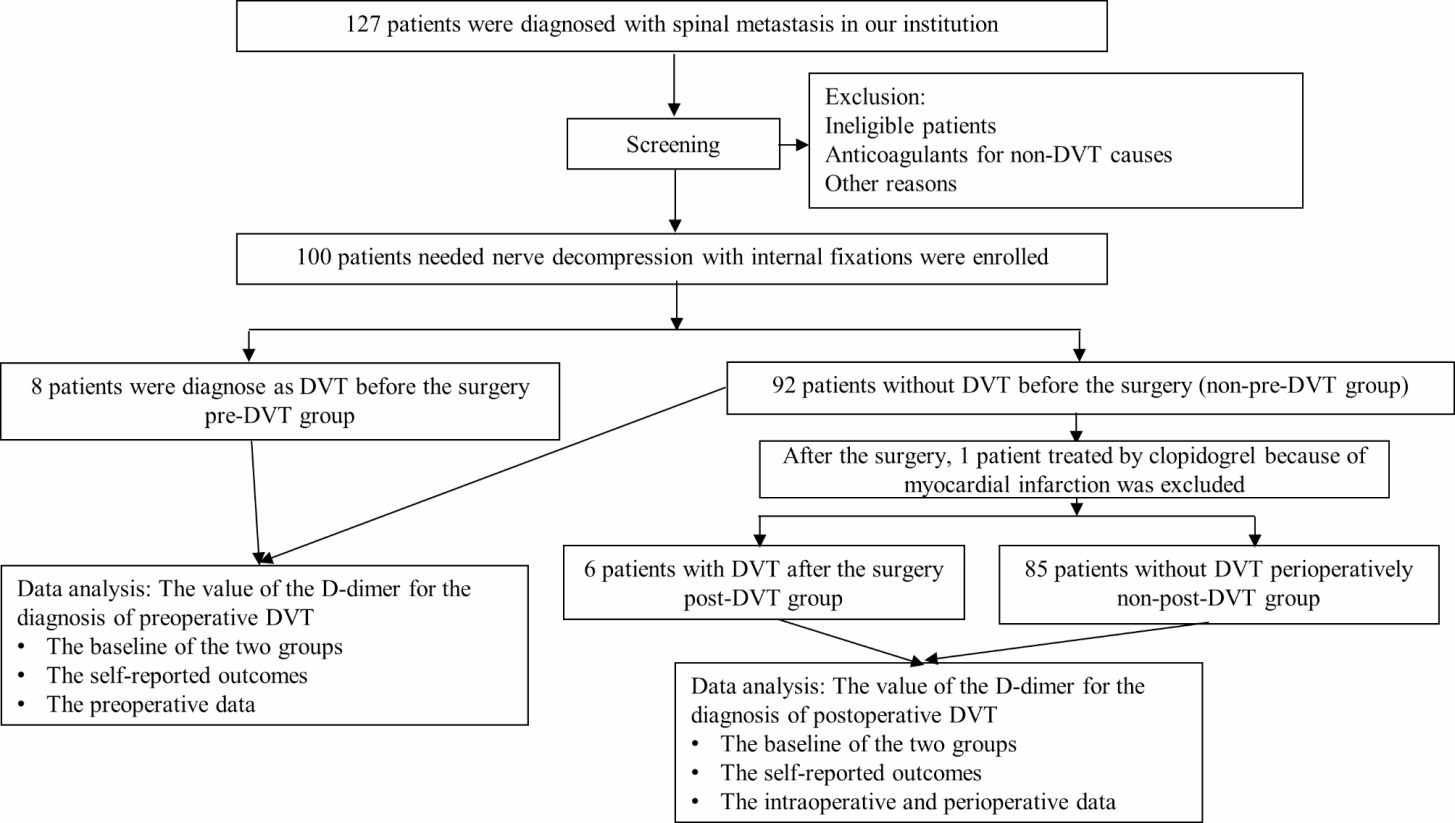
The flow chart of the study

A total of 127 patients were diagnosed with spinal metastases at our institution between October 2019 and October 2021, among whom 101 patients underwent nerve decompression with internal fixation. One patient had an artificial heart valve and required anticoagulant therapy. We explained the study process and the required tests to 100 patients, and informed consent was obtained from all the patients. Each patient was examined preoperatively using ultrasonography. If DVT was detected, patients with preoperative DVT (+) were enrolled in the pre-DVT(+) group, whereas the remaining patients were enrolled in the pre-DVT(-) group. After surgery, patients who were preoperative DVT(-) were examined again using ultrasonography. If DVT was detected after surgery, the patients were assigned to the post-DVT(+) group, and the remaining patients without DVT were enrolled into the post-DVT(-) group. To assess the value of D-dimer levels as a predictor of perioperative DVT, we compared the data of the pre-DVT(+) group with those of the pre-DVT(-) group and those of the post-DVT(+) group with those of the post-DVT(-) group. Figure 1 shows the flow chart of this study. This prospective cohort study was approved by the Ethics Committee of our institution and registered in the Chinese Clinical Trial Registry.

### Inclusion and exclusion criteria

Any patient with spinal metastasis with nerve compression symptoms requiring nerve decompression surgery with internal fixation was included, and spinal metastasis was diagnosed by biopsy of the tumor vertebra.

Patients with the following conditions were excluded, based on their medical history: coagulation dysfunction such as liver cirrhosis, hemophilia, hypovitaminosis and thrombophilia; hematological diseases such as thrombocytopenic purpura, leukemia, aplastic anemia and primary thrombocythemia; revision surgery, medications that affect coagulation function such as aspirin, clopidogrel, low molecular weight heparin and rivaroxaban.

### Clinical data

A visual analog scale (VAS; 0–10 scale) was used to evaluate back and radiating pain. Functional disabilities were measured using the Oswestry Disability Index (ODI) score and American Spinal Injury Association Impairment Scale (AIS). We determined the VAS, ODI, and AIS scores before surgery and on the day of discharge.

Patient information including age, sex, body mass index (BMI), tobacco use, comorbidities, history of chemotherapy or radiotherapy, type of primary tumor, Tomita score, and Tokuhashi score, as well as perioperative parameters including level(s) involved, operation time, blood loss, drainage volume, discharge days after surgery, transfusion, and preoperative transcatheter arterial embolization (TAE).

### Diagnosis of VTE and the D-dimer test

DVT was diagnosed using a duplex ultrasonographic assessment of both lower extremities. DVT scanning was done by a sonographer. Ultrasound machine was Mindary Resona R9 GE logic E9. The patient was in the supine position. The examination started from the common femoral vein in the inguinal region of both sides, passed through the bifurcation, and the upper and middle segments of the superficial femoral vein were scanned in cross section. The patient was then placed in the left lateral decubitus position, and a cross-sectional scan was performed from the popliteal vein down, including the posterior tibial vein and the intermuscular calf vein. Using doppler ultrasonography to observe vascular blood flow filling, in the face of poor filling trials on probe pressure vessel segment, observe whether venous lumen is fully closed. The location and size of the thrombus was identified. If PE was suspected or DVT was detected, the patient underwent multidetector computed tomographic pulmonary angiography. Blood tests, including D-dimer, hemoglobin (HB), platelet (PLT), and fibrinogen(FIB) tests, were performed twice: once before surgery and one day postoperatively. If a patient was VTE-positive, a vascular surgeon was consulted for VTE treatment. Patients without VTE received mechanical prophylaxis, including compression stockings and intermittent pneumatic compression devices, from the time of general anesthesia to postoperative ambulation.

### Statistical analyses

Statistical analyses included the calculation of descriptive statistics, including frequency counts for categorical data and dispersion (standard deviation and range) for continuous variables. We used the independent t-test for continuous parameters, Fisher’s exact test for dichotomous parameters, and the Mann-Whitney U test for AIS data. Receiver operating characteristic (ROC) curve analysis was performed to determine the optimal D-dimer threshold. The area under the curve (AUC) was calculated using the Hanley-McNeil nonparametric method. P values were considered statistically significant at *P* < 0.05. All the statistical analyses were performed using SPSS version 23 (IBM Corporation, Armonk, NY, USA).

## Results

### Patients

Between October 2019 and October 2021, 127 patients were diagnosed with spinal metastases at our institution. After screening, 100 patients who met the inclusion criteria were included in our study. (The full date of first registration was 08/10/2019). Eight patients were diagnosed with DVT before surgery and were included in the pre-DVT(+) group. Ninety-two patients without DVT were enrolled in the pre-DVT(-) group, among whom, six were found to have DVT postoperatively and were enrolled in the post-DVT(+) group. One patient was diagnosed with myocardial infarction one day after surgery and was treated with clopidogrel. Clopidogrel is one kind of anticoagulant drugs and can reduce the occurrence of DVT. That patient who received clopidogrel after surgery was different from the other patients in the coagulation status and was excluded from the postoperative and perioperative analysis of DVT prevalence rate; therefore, 85 patients were included in the post-DVT(-) group. The preoperative, postoperative, and perioperative rates were 8.0% (8/100), 6.6% (6/91), and 14.1% (14/99), respectively, and no patient presented PE.

### Pre-DVT(+) versus pre-DVT(-) group

Table 1 shows the preoperative data for the two groups. There were no significant differences in age, sex, body mass index (BMI), tobacco use, history of chemotherapy or radiotherapy, comorbidities, type of primary tumor, VAS score, ODI score, Tomita score, Tokuhashi score, levels involved, or preoperative HB, PLT, FIB, and D-dimer levels between the two groups. Only, the AIS of the pre-DVT(+) group was significantly worse than that of the pre-DVT(-) group.

**Table 1:** Pre-DVT(+) group versus pre-DVT(-) group

	pre-DVT(+)	pre-DVT(-)	*P* value
Number of cases	8	92	
Gender (male: female)	4:4	57:35	0.708
Age(years)	63.5 ± 6.1	59.5 ± 12.0	0.389
BMI	22.9 ± 4.6	23.8 ± 3.5	0.494
Number of smokers	0	11	0.593
history of radiotherapy	2	9	0.213
history of chemotherapy	3	23	0.425
Comorbidities			
Hypertension	3	30	1.000
Diabetes	0	8	1.000
CHD	0	3	1.000
COPD	0	1	1.000
Primary tumor			
Lung	2	35	
Liver	0	15	
Digestive tract	1	9	
Thyroid	0	2	
Breast	0	5	
Renal	0	10	
Others	5	16	0.112
Lower limb pain VAS (pre)	3.4 ± 2.9	3.1 ± 2.7	0.735
Thora-lumbar pain VAS (pre)	5.0 ± 1.7	4.9 ± 1.7	0.878
ODI (pre)	56.3 ± 14.0	44.8 ± 18.0	0.083
**AIS (pre)**			
** Grade A**	**1**	**0**	
** Grade B**	**0**	**2**	
** Grade C**	**3**	**10**	
** Grade D**	**1**	**6**	
** Grade E**	**3**	**74**	0.001*
Tomita Score	5.6 ± 2.3	6.2 ± 2.2	0.482
Tokuhashi Score	6.3 ± 3.9	7.7 ± 2.9	0.180
Level(s) involved	3.8 ± 1.2	3.8 ± 1.1	1.000
Preoperative			
HB(gram/liter)	121.6 ± 17.4	127.1 ± 16.7	0.382
PLT(10^9/ liter)	238.1 ± 59.2	214.4 ± 67.3	0.338
FIB(gram/liter)	351.4 ± 69.7	407.3 ± 138.5	0.263
D-dimer(milligram/liter)	5.8 ± 9.0	1.6 ± 2.4	0.231

* significant statistical difference between two groups

pre preoperative, BMI body mass index, CHD coronary heart disease, COPD chronic obstructive pulmonary disease, TAE transcatheter arterial embolization, VAS visual analog scale, ODI Oswestry Disability Index, AIS American Spinal Injury Association Impairment Scale, HB hemoglobin, PLT platelets, FIB fibrinogen

### Post-DVT(+)versus post-DVT(-) group

Table 2 shows the perioperative data for the two groups. In the blood test, the D-dimer level one-day postoperatively for post-DVT(+) group (17.6 ± 11.8 mg/L) was significantly higher than that of the post-DVT(-) group (5.0 ± 4.7 mg/L) (P value = 0.046). No significant differences were observed in the other blood test parameters. ODI scores and AIS reflect the patients’ neurological functions, and the post-DVT(+) group presented significantly worse findings than the post-DVT(-) group. The mean length of hospital stay after surgery until discharge for post-DVT(+) group (10.3 ± 4.1 days) was significantly longer than that of the post-DVT(-) group (6.5 ± 2.7 days) (P value = 0.001). There were no significant differences in age, sex, body mass index (BMI), tobacco use, history of chemotherapy or radiotherapy, comorbidities, type of primary tumor, VAS score, Tomita score, Tokuhashi score, operative time, estimated blood loss, drainage volume, levels involved, number of transfusions, or preoperative TAE between the two groups.

**Table 2:** Post-DVT(+) group versus post-DVT(-) group

	post-DVT(+)	post-DVT(-)	*P* value
Number of cases	6	85	
Gender (male: female)	4:2	53:32	1.000
Age(years)	56.2 ± 19.5	59.6 ± 11.5	0.686
BMI	24.1 ± 5.3	23.8 ± 3.4	0.840
Number of smokers	1	10	0.549
history of radiotherapy	1	8	0.475
history of chemotherapy	1	22	1.000
Comorbidities			
Hypertension	0	29	0.171
Diabetes	0	8	1.000
CHD	0	3	1.000
COPD	0	1	1.000
Primary tumor			
Lung	2	33	
Liver	0	14	
Digestive tract	1	8	
Thyroid	0	2	
Breast	0	5	
Renal	0	10	
Others	3	13	0.383
Lower limb pain VAS (pre)	2.3 ± 3.0	3.1 ± 2.7	0.528
Lower limb pain VAS (post)	1.2 ± 1.3	1.7 ± 1.5	0.427
Thora-lumbar pain VAS (pre)	5.3 ± 1.4	4.9 ± 1.8	0.540
Thora-lumbar pain VAS (post)	2.5 ± 0.5	2.6 ± 1.0	0.899
**ODI (pre)**	**60.0 ± 17.9**	**43.8 ± 17.7**	0.033*
**ODI (post)**	**40.0 ± 19.0**	**28.0 ± 13.6**	0.045*
**AIS (pre)**			
**Grade B**	**0**	**2**	
**Grade C**	**3**	**7**	
**Grade D**	**0**	**6**	
**Grade E**	**3**	**70**	0.017*
**AIS (post)**			
** Grade C**	**1**	**6**	
** Grade D**	**2**	**5**	
** Grade E**	**3**	**74**	0.029*
Tomita Score	7.3 ± 2.2	6.1 ± 2.2	0.205
Tokuhashi Score	6.0 ± 2.0	7.8 ± 2.9	0.137
Operative time (minutes)	175.0 ± 29.5	166.0 ± 30.7	0.489
Blood loss (milliliter)	466.7 ± 121.1	521.5 ± 420.1	0.752
Drainage volume (milliliter)	599.2 ± 502.9	381.0 ± 196.6	0.338
**Days after surgery until discharge**	**10.3 ± 4.1**	**6.5 ± 2.7**	0.001*
Level(s) involved	4.2 ± 1.2	3.7 ± 1.1	0.353
Number of Transfusion	3	35	0.691
Number of Preoperative TAE	2	19	0.619
Preoperative			
HB(gram/liter)	128.3 ± 16.2	127.1 ± 16.9	0.861
PLT(10^9/ liter)	218.3 ± 81.9	214.7 ± 66.9	0.900
FIB(gram/liter)	394.7 ± 90.1	407.2 ± 142.2	0.832
D-dimer(milligram/liter)	4.0 ± 7.3	1.5 ± 1.6	0.437
One-day postoperatively			
HB(gram/liter)	105.7 ± 8.4	109.6 ± 13.3	0.480
PLT(10^9/ liter)	191.8 ± 54.2	194.0 ± 70.0	0.942
FIB(gram/liter)	318.0 ± 62.8	382.3 ± 121.9	0.206
** D-dimer(milligram/liter)**	**17.6 ± 11.8**	**5.0 ± 4.7**	0.046*

* significant statistical difference between two groups

pre preoperative, post postoperative, BMI body mass index, CHD coronary heart disease, COPD chronic obstructive pulmonary disease, TAE transcatheter arterial embolization, VAS visual analog scale, ODI Oswestry Disability Index, AIS American Spinal Injury Association Impairment Scale, HB hemoglobin, PLT platelets, FIB fibrinogen

The area under the ROC curve for D-dimer level (post) that was used to distinguish between the two groups was 0.875 (P value = 0.002). Using ROC analysis, the optimum thresholds of the D-dimer level (post) was determined to be 9.51(mg/L). The sensitivity and specificity of the optimal threshold were 83.3% and 89.4%, respectively. The negative predictive value for the d-dimer level was 98.7%.

## Discussion

DVT and PE are well-known arthroplasty complications. Recently, VTE after spinal surgery has attracted increasing attention [1, 13–17]. The reported rates of PE after spinal surgery range from 0 to 7.6%, and those of DVT range from 0.27–31% [2–4, 18, 19]. We detected DVT in eight patients before surgery and in six patients after surgery, among 100 patients with spinal metastases. Perioperative ambulation has been reported as VTE risk factor [20]. Alexander et al. reported that the risk factors associated with the highest VTE incidence and odds ratios (ORs) were primary coagulation disorders (10.01%, OR 4.33) and extremity paralysis (7.49%, OR 2.96) in their database study [20]. The AIS score reflects the patient’s neurological function. In our study, the AIS scores of patients in the DVT (+) cohort were worse than that of those in the DVT (-) group, both preoperatively and postoperatively. If a patient has poor neurological function in the lower limbs, we should be alert for the occurrence of DVT.

D-dimer is a sensitive marker of thrombotic disease, and an increase in D-dimer levels indicates the existence of thrombi. D-dimer level is widely used to diagnose DVT and PE [8, 9, 11, 12, 21, 22], and Wei et al. reported that preoperative positive D-dimer levels were a significant risk factor for DVT onset after posterior lumbar surgery [21]. Furthermore, Ikeda et al. found that the cutoff value of preoperative D-dimer level was 1.4 mg/L for the diagnosis of postoperative DVT [11]. Yoshiiwa et al. reported in their retrospective study that a postoperative D-dimer level more than 10 mg/L was a risk factor for thromboembolic disease after spinal surgery. Their patients had spinal degeneration, spinal trauma, and tumors [9]. Additionally, Sudo et al. reported that plasma concentrations of D-dimer were significantly higher in patients with DVT than in those without DVT on postoperative days 4, 7, 10, and 14. In their study, D-dimer level greater than 17.7 mg/L on day 4 was suggested to be associated with DVT in patients after surgery [8]. Inoue et al. found that D-dimer is an effective marker of PE on postoperative days 3 and 7, with cutoff values of 8.2 mg/L and 10.8 mg/L, respectively, in patients following low-risk spine surgery [12]. In the present study, there was no difference in preoperative D-dimer level between the pre-DVT(+) and pre-DVT(-) groups before the surgery. As we focused on patients with spinal metastases, the malignant tumor itself was a risk factor for high D-dimer levels. Therefore, the preoperative D-dimer level may not be a perfect predictor of DVT in patients with spinal metastases before surgery, and we suggest that preoperative ultrasound examination should be performed for these patients. After surgery, the D-dimer level at day one of the post-DVT(+) group (17.6 ± 11.8 mg/L) was significantly higher than that of the post-DVT(-) group (5.0 ± 4.7 mg/L). Our cut-off value of D-dimer level (postoperatively) was 9.51(mg/L), which was similar to that reported in previous studies.

One limitation of our study was its short follow-up period, and delayed DVT or PE symptoms after hospital discharge were not observed. Another limitation is that we only performed D-dimer tests after surgery. Both limitations were due to the ethical rules, as we were prohibited from taking additional blood samples from patients and from performing follow-up examinations after discharge.

## Conclusion

This prospective study found that the prevalence rates of DVT pre-and postoperatively were 8.0% (8/100) and 6.6% (6/91), respectively, in patients with spinal metastases undergoing decompression with fixation. Thus, preoperative D-dimer level may not be a predictor of preoperative DVT. The cutoff value of the postoperative D-dimer was 9.51(mg/L), and the sensitivity and specificity for the optimum threshold were 83.3% and 89.4%, respectively. Poor neurological function is a high risk factor for perioperative DVT.

### Electronic supplementary material

Below is the link to the electronic supplementary material.

Additional file 1. Parameters data of all the patients

## Data Availability

Sequence data that support the findings of this study have been uploaded within supplementary information files.

## Abbreviations

VTE: Venous Thromboembolism
PE: Pulmonary Thromboembolism
DVT: Deep Venous Thrombosis
CT: Computed Tomographic
MRI: Magnetic Resonance Imaging
VAS: Visual Analog Scale
AIS: American Spinal Injury Association Impairment Scale
BMI: Body Mass Index
CHD: Coronary Heart Disease
COPD: Chronic Obstructive Pulmonary Disease
TAE: Transcatheter Arterial Embolization
HB: Hemoglobin
PLT: Platelets
PT: Prothrombin Time
APTT: Activated Partial Thromboplastin Time
FIB: Fibrinogen
